# Aspergillus Niger thermostable Cytosine deaminase-dextran conjugates with enhanced structure stability, proteolytic resistance, and Antiproliferative activity

**DOI:** 10.1186/s12866-023-02754-8

**Published:** 2023-01-10

**Authors:** Ashraf S. A. El-Sayed, Amgad M. Rady, Hossam Taha Mohamed, Nabila Zein, Marwa A. Yassin, Nabil Z. Mohamed, Abdallah Hassan, Mahmoud M. Amer, Reyad El-Sharakawy, Aya Ali El-Sharkawy, Nesma El-Sayed, Mostafa G. Ali

**Affiliations:** 1 Enzymology and Fungal Biotechnology Lab, Botany and Microbiology Department, Faculty of Science, Zagazig University, Zagazig, 44519 Egypt; 2 Faculty of Biotechnology, October University for Modern Sciences and Arts, Giza, 12451 Egypt; 3 Department of Zoology, Faculty of Science, Cairo University, Giza, 12613 Egypt; 4 Biochemistry Department, Faculty of Science, Zagazig University, Zagazig, 44519 Egypt; 5 Botany and Microbiology Department, Faculty of Science, Benha University, Benha, 13518 Egypt

**Keywords:** Cytosine deaminase, *Aspergillus Niger*, Dextran conjugation, 5-Fluorocytosine

## Abstract

Cytosine deaminase (CDA) is a prodrug mediating enzyme converting 5-flurocytosine into 5-flurouracil with profound broad-range anticancer activity towards various cell lines. Availability, molecular stability, and catalytic efficiency are the main limiting factors halting the clinical applications of this enzyme on prodrug and gene therapies, thus, screening for CDA with unique biochemical and catalytic properties was the objective. Thermotolerant/ thermophilic fungi could be a distinctive repertoire for enzymes with affordable stability and catalytic efficiency. Among the recovered thermotolerant isolates, *Aspergillus niger* with optimal growth at 45 °C had the highest CDA productivity. The enzyme was purified, with purification 15.4 folds, molecular mass 48 kDa and 98 kDa, under denaturing and native PAGE, respectively. The purified CDA was covalently conjugated with dextran with the highest immobilization yield of 75%. The free and CDA-dextran conjugates have the same optimum pH 7.4, reaction temperature 37 °C, and *pI* 4.5, and similar response to the inhibitors and amino acids suicide analogues, ensuring the lack of effect of dextran conjugation on the CDA conformational structure. CDA-Dextran conjugates had more resistance to proteolysis in response to proteinase K and trypsin by 2.9 and 1.5 folds, respectively. CDA-Dextran conjugates displayed a dramatic structural and thermal stability than the free enzyme, authenticating the acquired structural and catalytic stability upon dextran conjugation. The thermal stability of CDA was increased by about 1.5 folds, upon dextran conjugation, as revealed from the half-life time (*T*_*1/2*_). The affinity of CDA-conjugates (*K*_*m*_ 0.15 mM) and free CDA (*K*_*m*_ 0.22 mM) to deaminate 5-fluorocytosine was increased by 1.5 folds. Upon dextran conjugation, the antiproliferative activity of the CDA towards the different cell lines “MDA-MB, HepG-2, and PC-3” was significantly increased by mediating the prodrug 5-FC. The CDA-dextran conjugates strongly reduce the tumor size and weight of the Ehrlich cells (EAC), dramatically increase the titers of Caspase-independent apoptotic markers PARP-1 and AIF, with no cellular cytotoxic activity, as revealed from the hematological and biochemical parameters.

**Supplementary Information:**

The online version contains supplementary material available at 10.1186/s12866-023-02754-8.

## Introduction

Cytosine deaminase (CDA, EC 3.5.4.1), is an enzyme hydrolyzes the carbon-nitrogen bond of cytosine into uracil and ammonia [1]. Cytosine deaminase has been reported to be produced from different fungal and bacterial isolates and is absent in mammalian tissues [2–4]. Cytosine deaminase has recently received much attention for its profound implementationin mediating the prodrugs of cancer chemotherapy. The rationality of using CDA in cancer therapy elaborates from its affinity to convert the inactive prodrug 5-fluorocytosine into the active drug 5-fluorouracil (5-FU) [5–7]. 5-Fluorouracil (5-FU) has been widely used as a powerful anticancer drug for various types of tumor cell lines, however, the shortage of its biological half-life time and lacking the selectivity, are the main challenges that-impedes their further clinical applications [8].

5-FU has been approved by the FDA as an antifungal drug that is efficiently capable of crossing the blood-brain barrier, as an orally bioavailable compound [2], especially with the emergence of tumor resistance phenomena to the current chemo and radiotherapies [9, 10]. Thus, the application of the prodrug 5-FC, which subsequently activated into 5-FU under control by CDA is one of the most reliable approaches for minimizing and targeting the toxic effect of 5-FU to only diseased parts. This strategy can increase the therapeutic efficiency and pharmacological potentiality of 5-FU. Thus, searching for enzymes with higher potency for mediating the activation of the prodrug is the current challenge [11]. CDA has been characterized from various bacteria [12, 13], and fungi [13–15], and its activity was validated as an efficient anticancer agent by mediating the conversion of prodrug 5-fluorocytosine with efficiency against several cancer cells. Bacterial CDA has received great interest, over eukaryotic enzyme sources, despite the higher activity and catalytic efficiency of the eukaryotic derived-CDA, than the bacterial enzyme. However, the major challenge for this enzyme are the higher antigenicity, lower thermal stability and short life span [16], lower structural/ thermal stability, and catalytic efficiency, which limits the further clinical applications of this enzyme [17]. In clinical trials, CDA is one of the strategies which developed rapidly in tumor gene therapy, and its anticancer efficiency has been extensively emphasized for different cancer types [18]. Recently, yeast *cda* was cloned in Vaccinia virus Guang 9 (VG9) for mediating the drug 5-FU from the prodrug 5-FC, displaying a powerful efficiency for colorectal cancer cell therapy in vitro and in vivo [3]. Combination of Temozolomide andmesenchymal stem cells expressing the *cda* gene, followed by the addition of 5-FC strongly suppress glioma cell proliferation [4]. Cytosine deaminase had been purified from yeasts displaying higher catalytic properties than bacterial ones but, has a lower thermal stability than the bacterial CDA [12, 19]. Thermophilic/thermotolerant fungi could have a robust enzymatic system with higher thermal/structural stability and catalytic efficiency [5–11, 20]. Thus, CDA from thermophilic/thermotolerant fungi could have unique kinetic properties and structural stability. The objective of this work was to purify and biochemically characterize CDA from various thermophilic/ thermotolerant fungal isolates, in addition, to conjugation of CDA with dextran as a biocompatible polymer to increase the enzyme conformational stability and catalytic efficiency.

## Results

### Screening and identification of the potent CDA-producing thermophilic fungal isolates

**Fig. 1:**
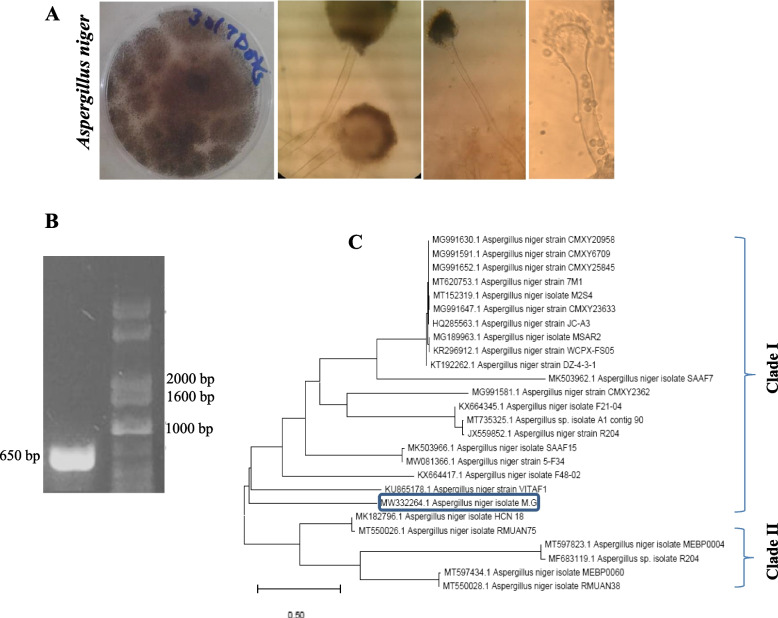
Morphological and molecular identification of the potent thermotolerant CDA-producing fungi grows on PDA at 45 °C for 7 days. A, Macro and micro-morphological features of *A. niger* on PDA, conidial heads by light microscope 100 x. B, PCR amplicon of the ITS region of *A. niger* in reference to DNA ladder (1 kb Ladder, Cat#. MD113–01). C, Molecular phylogenetic analyses of *A. niger* ITS sequence were conducted by Maximum Likelihood Model, the FASTA sequences were aligned with Clustal W, and the phylogenetic tree was constructed by MEGA X Software package with 250 bootstrap replication

Twenty-six fungal isolates were purified Czapek’s-Dox agar media after 7 days of incubation at 45 °C (Table S1). The intracellular crude proteins were extracted and the CDA activity was determined. The highest yield of CDA was detected for *A. niger* 2 (54.6 μmol/mg/min), *A. fumigatus* 1 (47.7 μmol/mg/min), and *A. flavus* 1 (47.7 μmol/mg/min). Based on the morphological identities, the purified fungal isolates were identified to their species level using the universal identification keys on PDA and Czapek’s-Dox media. The colonies of the most potent CDA-producer had black conidiophores with reverse pale to bright yellow, the conidiophores arise from hyphae with heavy smooth walls, with spherical heads, and stigma covers the entire surface of the vesicle (Fig. 1) that were identical to *Aspergillus niger*. The morphological identity of *A. niger* was confirmed from the ITS region sequence. The PCR amplicon of the ITS region (~ 650 bp) was sequenced and gave a 99.5% similarity with the corresponding database-deposited isolates of *A. niger*. The ITS sequence of *A. niger* EFBL2022 was deposited into the Genbank with accession # MW332264.1. From the phylogenetic analysis, *A. niger* MW332264.1 gave 99.5% similarity with *A. niger* KU865178.1, KX664417.1, MW081366.1, and MK503966.1, with zero E value (Fig. 1).

### Purification and molecular subunit structure of *a. niger* CDA

**Fig. 2:**
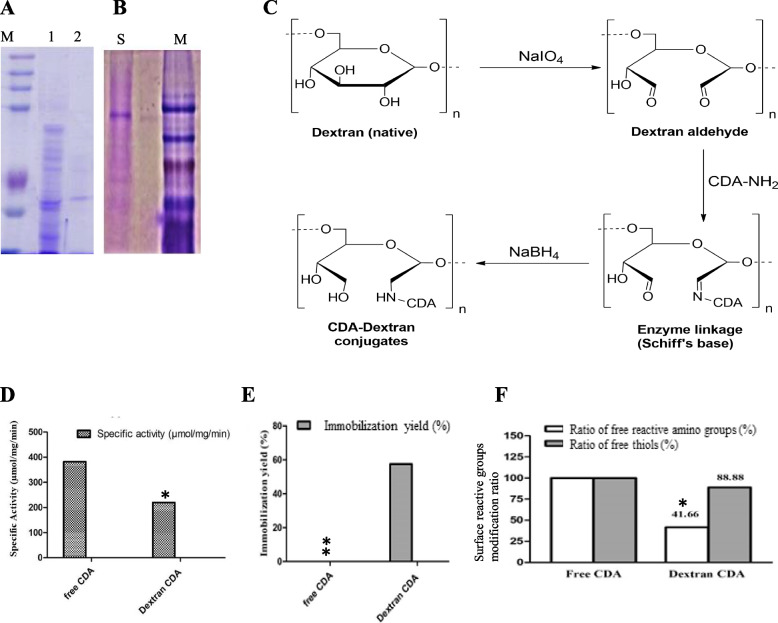
Purification and dextran conjugation of CDA from *A. niger.* The enzyme was extracted from the mycelia of *A. niger*, and purified by gel-filtration and ion-exchange chromatography. The molecular homogeneity of the purified enzyme was assessed. A, SDS-PAGE profile of purified CDA (M, protein ladder, Blue Plus Protein Maker, Cat # DM101, 14–100 kDa, lane 1 is the crude protein, lane 2 is the purified enzyme). B, Native-PAGE of the purified CDA from *A. niger.* C, Scheme of dextran activation and covalent conjugation of CDA, dextran was activated by sodium periodate forming reactive aldehyde groups to form Schiff base with the CDA surface reactive amino groups, with subsequent reduction by sodium borohydride. Specific activity (D), immobilization yield (E), and modification of surface reactive ε-amino groups of lysine residues and reactive amino groups of amide amino acids (F). The significant *p-values < 0.032, and highly significant **p-values < 0.002, were calculated by Student’s *t*-test to the free CDA in response to Dextran conjugation

The fungal isolate was grown on PDB, the mycelia were collected, and the intracellular crude proteins were extracted, fractionally concentrated by dialysis, and purified by gel-filtration and ion-exchange chromatographic approaches. The most active, molecular homogenous fractions from the Sephadex-G100 column were further purified by the DEAE-Sepharose column. From the overall purification profile (Table 1), the activity of purified *A. niger* CDA has increased by 4.19 and 15.4 folds upon purification by gel-filtration and ion-exchange chromatography, respectively, with an overall yield of 40.2%. The molecular mass and subunit structure of the purified CDA from *A. niger* was checked by native-PAGE and SDS-PAGE profile (Fig. 2), with approximately 100 kDa, and a single proteinaceous band of 48 kDa, respectively. Thus, from the native and SDS-PAGE, the purified *A. niger* CDA has two identical subunits, i.e. homodimeric identity.

**Table 1:** Overall purification profile of cytosine deaminase from *A. niger*

Step	Volume (ml)	Total activity (U)	Total protein (mg)	Specific activity (μmol/mg/min)	Recovery (%)	Purification fold
**Crude Enzyme**	37	28,672.68	524.82	54.63	100.00	1.00
**Dialysis**	4	2194.84	20.20	108.68	7.65	1.99
**Sephadex G100**	9	7107.84	31.08	228.70	24.79	4.19
**DEAE-Sepharose**	10	11,475.36	13.58	844.93	40.02	15.47

### CDA-dextran conjugation and characterization

To enhance the structural stability and catalytic efficiency of the purified CDA, the enzyme was conjugated with the activated dextran, via an aldimine linkage of enzyme primary amino groups and reactive aldehyde groups of dextran (Fig. 2). Different molar ratios of CDA and activated dextran were tested, and the enzyme activity and immobilization yield were assessed. Obviously, the activity of CDA and immobilization yield was increased with increasing enzyme molar ratio, regarding dextran concentration. The highest activity of CDA-dextran conjugates (219.9 μmol/mg/min) was reported upon using 0.21 mM CDA to 20 mM activated dextran, with a conjugation yield of 57.54% (Table 2). The surface lysine residues of CDA form a Schiff base of glucose residues, so, the optimization of the conjugation ratio of enzyme and dextran is a very limiting step. The negative effect on the activity of CDA upon conjugation with dextran could be due to the steric hindrance of the enzyme active sites by the dextran molecule hindering the enzyme-substrate complex formation. Thus, 0.203 mM of CDA to 20 mM activated dextran has the lowest steric hindrance for enzymatic substrate active site complex formation. The specific activity of CDA conjugated with Dextran was 219.9 compared to 382.3 μmol/mg/min of free CDA (Fig. 2) with an immobilization yield of 57.54%. The residual free reactive amino groups of CDA-Dextran conjugates was 41.6% compared to the free CDA, ensuring the implementation of 58.4% of the surface amino groups of CDA in covalent binding with the dextran-activated aldehyde group (Table 3). The modification ratio of the surface-free thiols of cysteine residues of CDA was 11.2% upon dextran conjugation, suggesting the slight interaction of the chemical modifiers with the enzyme surface thiols (Table 3). Thus, the CDA conjugation with dextran was mainly due to the interaction with amino groups of lysine residues, unlike, the lower interaction of surface amide amino acid, and the surface thiols.

**Table 2:** Different kinetic parameters in response to different molar ratio of dextran with purified cytosine deaminase of *A. niger*

Dextran (mM)	CDA (mM)	Specific activity (μmol/mg/min)	Conjugation yield (%)
–	0.067	376.61	–
	0.135	319.91	–
	0.203	382.23	–
20	0.067	189.22	50.24
	0.135	146.45	45.77
	0.203	219.97	57.54

**Table 3:** Enzyme surface modification of free amino and thiols groups

Enzyme	Reactive amino groups (%)		Surface thiols (%)	
	Ratio of free reactive amino groups (%)	Degree of modification (%)	Ratio of free thiols (%)	Degree of modification (%)
**Free-CDA**	100	–	100	–
**Dextran-CDA**	41.66	58.34	88.88	11.12

### In vitro proteolysis of the free and CDA-dextran conjugates

**Fig. 3:**
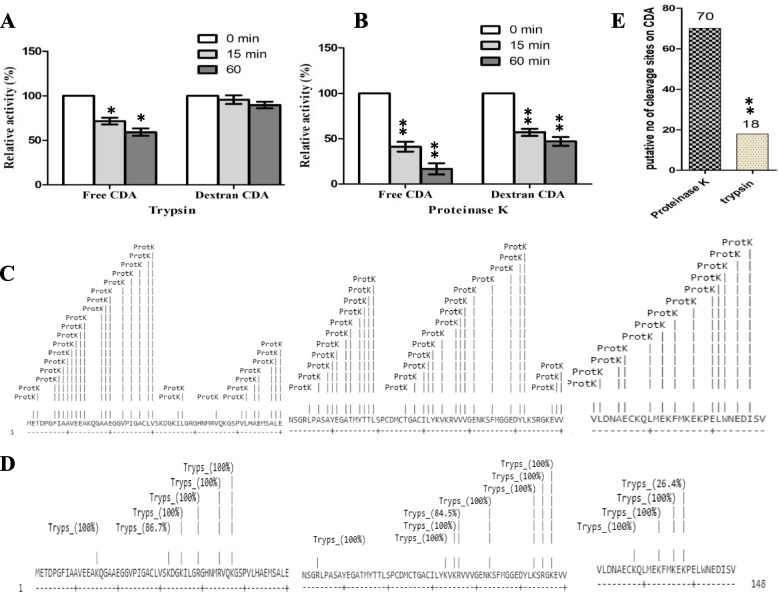
Catalytic activity and structural stability of free *A. niger* and CDA-Dextran conjugates in response to proteolytic cleavage by proteinase K and trypsin. The relative activity of the free and Dextran-ADI conjugates in response to digestion by Trypsin (A) and proteinase K (B) for 15 and 60 min at 37 °C. The putative proteolytic map of *A. niger* CDA peptide fragments in response to proteinase K (C) and trypsin (D) by ExPASy (https://web.expasy.org/peptide_cutter). E, the putative number of cleavage sites of CDA in response to trypsin and proteinase K. The significant *p-values < 0.05, and highly significant **p-values < 0.005, were calculated by Student’s *t*-test to the free CDA and Dextran-CDA conjugates in response to Trypsin and proteinase K treatment

The effect of dextran conjugation on blocking the surface proteolytic recognition sites of CDA was assessed. The enzymes were incubated with proteinase K and trypsin (10 μmol/mg/min), and their residual activities were determined, intervally. The CDA-dextran conjugates displayed more resistance to proteinase K and trypsin by 3.0 and 1.5 folds for 60 min, respectively, compared to free CDA, suggesting the shielding of recognition proteolytic sites on the enzyme surface upon dextran conjugation (Fig. 3). The free and CDA-Dextran conjugates retain 16.7 and 57%, respectively, of their initial activities in response to proteinase K for 60 min. However, in response to trypsin, the free and CDA-Dextran conjugates retain about 59.24 and 89.73%, respectively after 60 min (Fig. 3). Remarkably, the sensitivity of the free and CDA-Dextran conjugates to proteolysis by proteinase K was higher than trypsin by 3.6 and 1.9 folds suggesting the frequent multiple recognition sites of proteinase K on CDA surface than trypsin. This result was theoretically confirmed from the in silico prediction of proteolytic sites by peptide cutter (ExPASY, bioinformatics Resource Portal) as shown in Fig. 3. Practically, the total number of proteolytic cleavage surface sites of CDA was found to be 70, and 18 recognition sites for proteinase K and trypsin, respectively. So, the higher proteolysis of CDA upon incubation with proteinase K for 60 min is reasonable, since this enzyme attacks multiple recognition sites on the CDA surface. Practically, the free CDA is highly resistant to cleavage by trypsin than proteinase K which might be due to the lower frequency of recognition sites for trypsin.

### Biochemical properties of free CDA and CDA-dextran conjugates

**Fig. 4:**
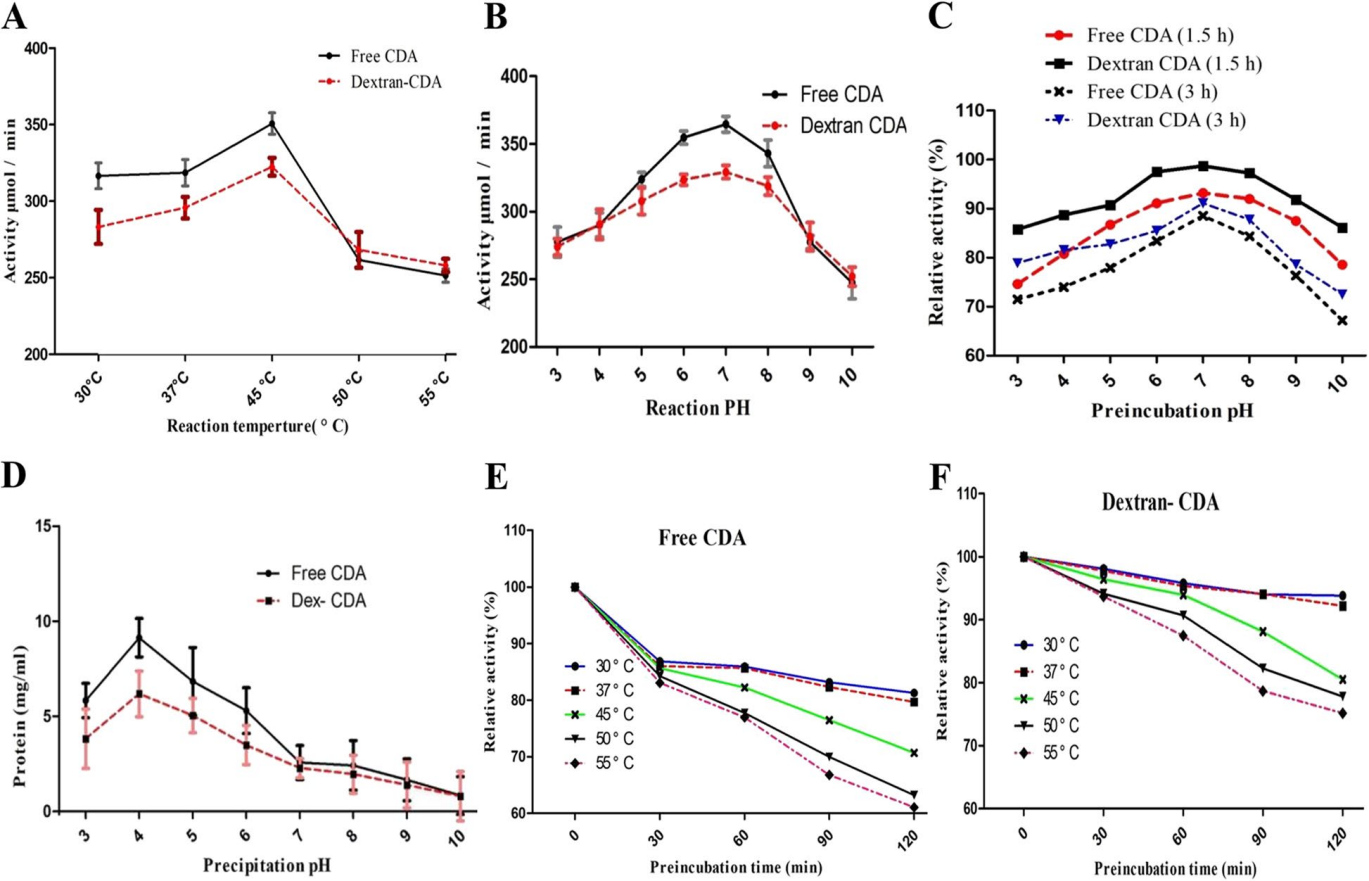
Biochemical properties of the free and CDA-Dextran conjugates from *A. niger*. The effect of reaction temperature (A), reaction pH (B), pH stability (C), and pH precipitation (D) of the free and CDA-dextran conjugates from *A. niger.* The thermal stability profile of free CDA (E) and CDA-dextran conjugates (F)

The biochemical properties “thermal stability, pH stability, reaction temperature, and reaction pH” of the enzyme were estimated. From the profile of reaction temperature (Fig. 4), the activity of free CDA and CDA-Dextran conjugates was relatively similar, with a maximum value at 37 °C. With the higher incubation temperature, the activity of CDA was slightly reduced suggesting the enzyme denaturation and disassembly of the enzyme subunits. The thermal kinetic parameters of the free and CDA-dextran conjugates were reported in Table 4. From the results, the half-life time (*T*_*1/2*_) of CDA was increased by 1.5 folds for each temperature, upon dextran conjugation. At 30 °C, the half-life time (*T*_*1/2*_) of CDA-dextran conjugates (48.63 h) was increased by about 1.5 folds compared to the free CDA (32.5 h), with a significant decreasing in the thermal denaturation rate of CDA-Dextran conjugates (0.043 min^− 1^) by 3.5 folds compared to the free enzyme. At 37 °C, the *T*_*1/2*_ of the free CDA (29.38 h) was increased by about 1.4 folds upon conjugation with dextran (39.27 h). The profile of thermal stability of the free and CDA-dextran conjugates at 30, 37, 45, 50, and 55 °C was shown in Fig. 4. Noticeably, from the thermal stability profile, the structural and tertiary orientation of the CDA was strongly increased upon conjugation with dextran. From these results, the thermal and catalytic stability of CDA has strongly increased upon conjugation with dextran by ~ 1.07, 1.35, and 1.28 folds at 45, 50, and 55 °C, respectively. The effect of pH on the enzyme reaction of both free and CDA-Dextran conjugates was studied using different buffers giving a pH range of 3.0–10.0. From the results (Fig. 4), the free and CDA-dextran conjugates showed the same response to reaction pH, maximum activity at pH 6.5–7.0 with a significant reduction to their activities at acidic pH 3.0 and alkaline pH 10.0. The pH precipitation of the enzyme was assessed by incubating the free and CDA-Dextran conjugates at the same concentration for 24 h at 4 °C, centrifugation, and measuring the amount of precipitated protein. The result shows that the highest protein precipitation was achieved at pH (4.0), for the free and Dextran-conjugated CDA, negating the effect of pH on CDA ionic charge in response to dextran conjugation. The pH stability of the free and CDA-Dextran conjugates was assessed by pre-incubating at different pHs (3.0–10.0) for 3 h at 4 °C, then their residual activities were measured. The free and CDA-dextran conjugates have the same pH stability paradigm in response to different pHs (Fig. 4), with maximum activity at pH 6.0–8.0. However, the CDA-Dextran conjugates had higher catalytic stability than the free one, assuming the positive effect of dextran conjugation on the enzyme’s structural stability. So, from the different profiles of pH on enzyme reaction, precipitation, and stability, it clearly appeared that CDA conjugation with dextran does not affect the enzyme structure and ionic state.

**Table 4:** Thermal kinetic parameters of *A.niger* free and Dextran-CDA conjugates

Temp.(°C)	Free- CDA		Dextran- CDA		Stabilizing Ratio (%)
	*T*_*1/2*_ (h)*	*Kr* (min) **	*T*_*1/2*_ (h)	*Kr* (min)	
30	32.51	0.152	48.63	0.043	1.50
37	29.38	0.155	39.27	0.048	1.34
45	12.95	0.200	13.92	0.063	1.07
50	9.27	0.250	12.54	0.098	1.35
55	8.61	0.260	11.07	0.134	1.28

*Half-life time (T_*1/2*_) was expressed by time, which the enzyme retains 50% of its initial activity by preheating without substrate at each temperature degree

** Thermal denaturation rate (*K*_*r*_) was expressed by the logarithmic decreasing of enzyme activity with the time at each temperature. It described by the first-ordered kinetic model; ln (At/A0) = − K_r_ T_m_ where A_0_ and at are the specific activity of CDA at zero and t time

### Active site mapping of CDA by the amino acid suicide analogues and inhibitors

Enzyme active sites mapping by chemical inhibitors has been used frequently to predict the amino acids residues implemented with the enzyme catalytic efficiency and structural stability [21–25]. Prior to the addition of chemicals, the enzymes were de-metalized by dialysis with Tris-HCl buffer (50 mM, pH 7.0) of 1.0 mM EDTA. The relative activity of the demetallized free and CDA-Dextran conjugates was 60.7 and 74.8%, respectively, compared to their initial activities, assuming the metallo-proteineous identity of this enzyme. From the results (Table 5), an obvious fluctuation in the enzyme activity was reported in response to each compound, assuming the versatile effect of these compounds on enzyme catalytic identity. The activities of free and CDA-dextran conjugates were restored upon the addition of K^+^ ions, followed by Fe^2+^ and Ba^2+.^ While, the residual activity of the enzymes was approximated by 40–45% in presence of Ca^2+,^ Hg^2+,^ Al^3+,^ and Na^+^, that being similar to the activity of apo-CDA, assuming the negligible effect of these cations on CDA catalytic activity. Complete restoring the activity of CDA on the monovalent cations K^+,^ ensuring the metalloproteinic identity, using K^+^ as a cofactor.

**Table 5:** Relative activity of both free and Dextran-CDA conjugates in response to different general blocker and inhibitors

Inhibitors and amino acid suicide analogues.	Relative activity % free CDA	Relative activity % of CDA-Dextran conjugates
Control	100.00	100.00
DTNB	8.71	46.71
H_2_O_2_	6.22	60.53
MBTH	24.90	55.26
Hydroxylamine	9.54	37.50
Guanidine thiocyanate	16.60	41.45
Iodoacetamide	8.30	41.45
Apo-CDA	60.70	74.80
FeCl3	87.97	82.24
KCl	92.95	90.13
CaCl2	79.67	59.21
CuSo4	34.02	61.84
BaCl2	77.59	84.21
HgCl2	38.17	46.97
K2Cr2O7	43.57	41.05
AlCl3	66.39	77.63
NaCl	33.86	47.37

The amino acid suicide analogues guanidine thiocyanate, iodoacetamide, hydroxylamine, DTNB, MBTH, and H_2_O_2_ have been used to assess the chemical identity of the active sites and conjugation identity with dextran. Overall, CDA conjugation with dextran had a noticeable stabilizing effect on the enzyme’s tertiary and catalytic structures. As shown from the results (Table 5), the activity of CDA-dextran conjugates was higher than the free one in response to different inhibitors, ensuring the positive effect of dextran residues on stabilizing the enzyme tertiary structure and protecting the surface reactive groups. The activity of CDA-Dextran conjugates was higher than free CDA by about 10 folds in response to H_2_O_2_. The activity of free CDA was reduced by 2 folds compared to CDA-dextran conjugates, in response to MBTH, as a specific reactive inhibitor to the surface primary amino groups. The free enzyme and CDA-dextran conjugates retain 9.5 and 37.5% of their initial activities, respectively, in response to hydroxylamine, revealing the stabilizing effect of dextran moieties on surface reactive lysine residues. The activity of CDA-dextran conjugates was higher than the free CDA by about 5.4 folds in response to DTNB, confirming the sensitivity of free CDA, and shielding the surface L-cysteine residues from oxidation by DTNB. Remarkably, conjugation f CDA with dextran strongly shields the surface reactive lysine and cysteine residues of the enzyme from the oxidation by the tested compounds.

### Kinetics and catalytic properties of native and dextran-ADI conjugates

The affinity of free and CDA-conjugates to deaminate the free amino acids regarding cytosine as standard substrate was assessed based on the amount of released ammonia. From the results, the conjugation of dextran had little effect on the catalytic properties of CDA by shifting their catalytic properties toward various free amino acids. From the results (Table S2), the free and CDA-Dextran conjugates have undetectable activity towards the experimented amino acids, normalizing to cytosine as substrate. Interestingly, the free and CDA-dextran conjugates have the same catalytic efficiency towards the aromatic amino acids namely L-tryptophan, phenylalanine, and tyrosine, ranging from 4.0–7.0% normalizing to cytosine as authentic substrate.

The kinetic properties of the free and CDA-dextran conjugates towards cytosine and 5-fluorocytosine were assessed based on the released uracil and 5-fluorouracil (Table 6). Both free and CDA-Dextran conjugates displayed a significant affinity to deaminate cytosine and 5-fluoro-cytosine. The affinity (*K*_*m*_) of the free and CDA-dextran conjugates for cytosine deamination was 0.36 and 0.77 mM, while was 0.22 and 0.15 mM for 5-fluorocytosine deamination, respectively. So, upon dextran conjugation, the CDA deaminating affinity to free 5-fluorocytosine was increased by ~ 1.5 fold. The catalytic efficiency (*K*_*cat*_*/K*_*m*_) of CDA-dextran conjugates (0.6 mM^− 1^ s^− 1^) was increased by~ 1.43 folds, compared to free CDA (0.42 mM^− 1^ s^− 1^) for deaminating 5-fluorocytosine. Interestingly, the catalytic efficiency of CDA-Dextran conjugates (0.60 mM^− 1^ s^− 1^) for deamination of 5-fluorocytosine was higher than cytosine (0.12 mM^− 1^ s^− 1^) by about 5 folds.

**Table 6:** Kinetic parameters of free and Dextran-CDA conjugates from *A. niger*

Substrate	Free CDA				Dextran- CDA			
	K***m*** (mM)	V***max*** (μmol/mg/ min)	K***cat*** (S^**−1**^)	K***cat***/K***m*** (mM^**−**^1 s^**−1**^)	K***m*** (mM)	V***max*** (μmol/mg/min)	K***cat*** (s^**−1**^)	K***cat***/K***m*** (mM^**−1**^S^**−1**^)
Cytosine	0.36	333.33	0.077	0.22	0.77	384.61	0.089	0.12
5-Flurocytosine	0.22	400.00	0.093	0.42	0.15	384.61	0.089	0.60

### In vitro anticancer activity

**Fig. 5:**
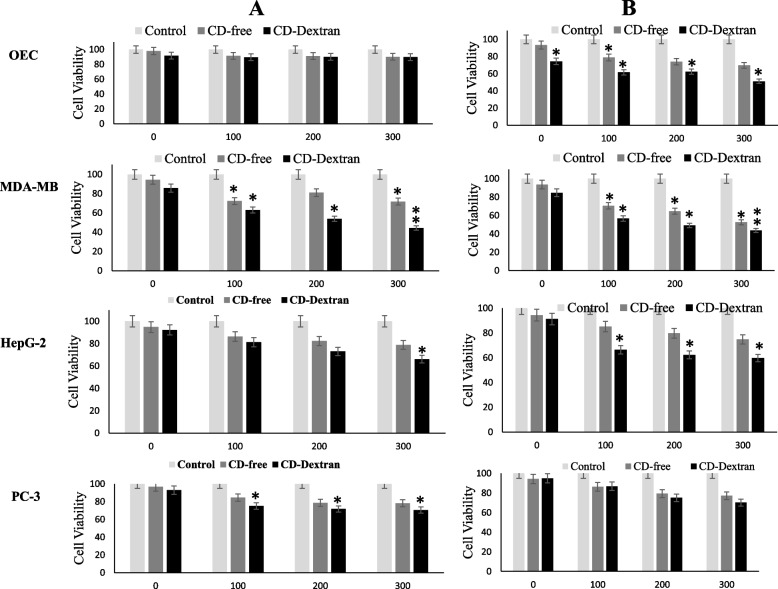
The antiproliferative activity of free and CDA-Dextran conjugates mediating the prodrug 5-FC towards the cell lines OEC, MDA-MB, HepG-2, and PC-3. Different concentrations of the prodrug 5-FC (0, 100, 200, and 300 ng/ml) were amended to the wells of the microtiter plate, then the free and CDA-dextran at 100 μmol/mg/min (A) and at 200 μmol/mg/min (B) were added, and the cellular viability was determined by MTT assay. The viability of the MDA-MB, HepG-2, and PC-3, in addition to the normal cells OEC was determined. The significant *p-values < 0.1, and highly significant **p-values < 0.01, were calculated by Student’s *t*-test to the activity of the free CDA and Dextran-CDA conjugates towards the lines “OEC, MDA-MB, HepG-2, and PC-3”

The antiproliferative activity of the native and CDA-Dextran conjugates was evaluated towards different tumor cell lines; liver carcinoma (HepG-2), breast carcinoma (MDA-MB-231), and prostate cancer (PC-3) regarding the Oral Epithelial cells (OEC) by the MTT assay. From the results (Fig. 5), the prodrug system of 5-FC-CDA had no obvious effect on the cellular processes and biological growth of the OEC normal cells, compared to the dramatic effect on HepG-2, MDA-MB-231 and PC-3 cell lines, in response to the free CDA and CDA-dextran conjugates at 100 μmol/mg/min. Remarkably, the 5-FC-CDA system displayed significant activity against the MDA-MB-231, followed by HepG-2 and PC-3 cell lines, as revealed from the viability pattern. The viability of MDA-MB-231 was reduced by about 50 and 60% in response to free-CDA and CDA-dextran conjugates (100 μmol/mg/min), respectively, at 300 μg/ml 5-FC. At the different concentrations of 5-FC, the antiproliferative activity of CDA- dextran conjugates was higher than the free CDA by about 1.8 folds, against the MDA-MB-231. As well as, the viability of HepG-2 and PC-3 cells was reduced by about 40–50% in response to the free CDA, and CA-Dextran conjugates, at concentrations of 200–300 μg/ml of 5-FC. Obviously, with the higher concentrations of 5-FC prodrug and CDA activity, a noticeable reduction in the viabilities of tested cell lines was observed, assuming the concentration-dependent manner of the 5-FC-CDA system on suppressing the cellular process of tumor cells.

### In vivo pharmacokinetics of native and CDA-dextran conjugates

**Fig. 6:**
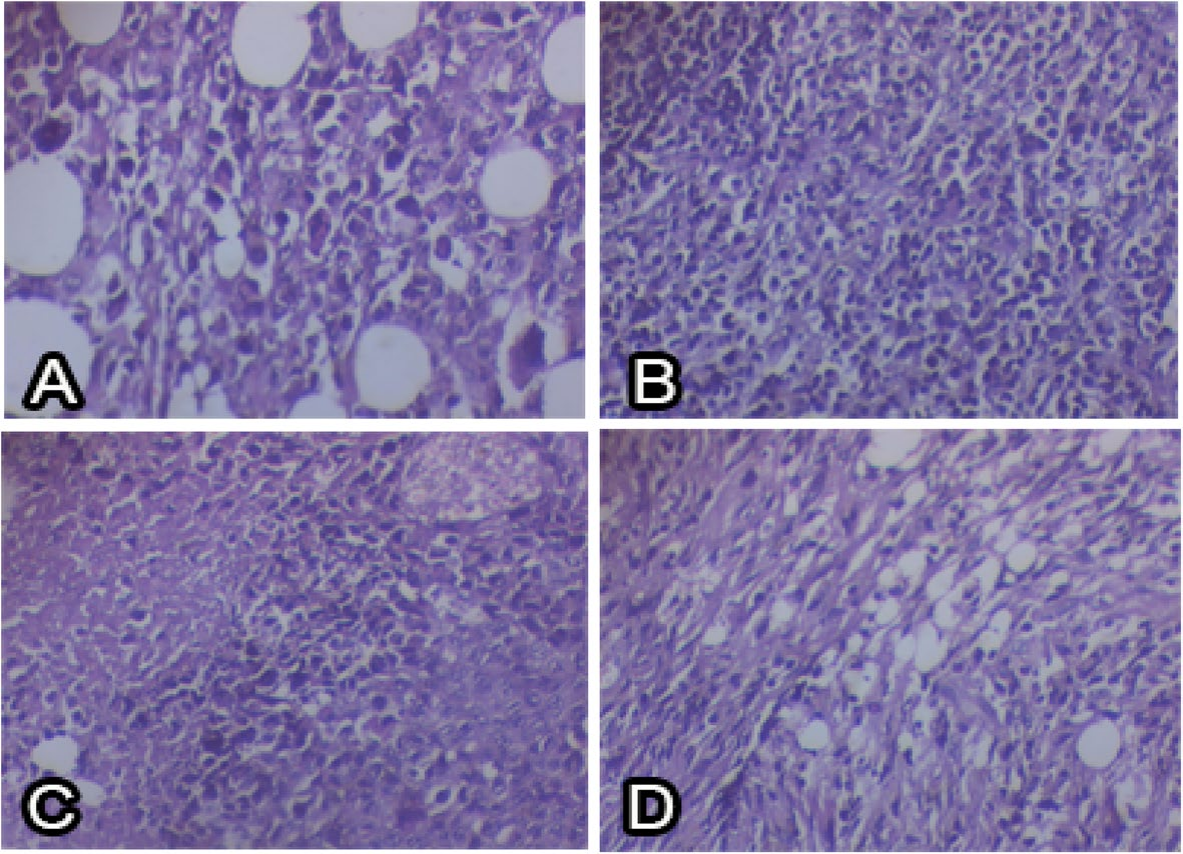
Histopathological sections of EAC solid tumor from the mice. A, positive control, most of the tumor cells showed apoptosis and necrosis with high proliferation and bizarre giant cells. B, Negative control showed marked necrosis and bizarre giant cells. C, Free CDA-treated mice showed moderate proliferation and apoptosis. D, CDA-dextran conjugates showed a decrease in malignant cells, proliferation, fibrosis, and apoptosis using H and E staining

**Fig. 7:**
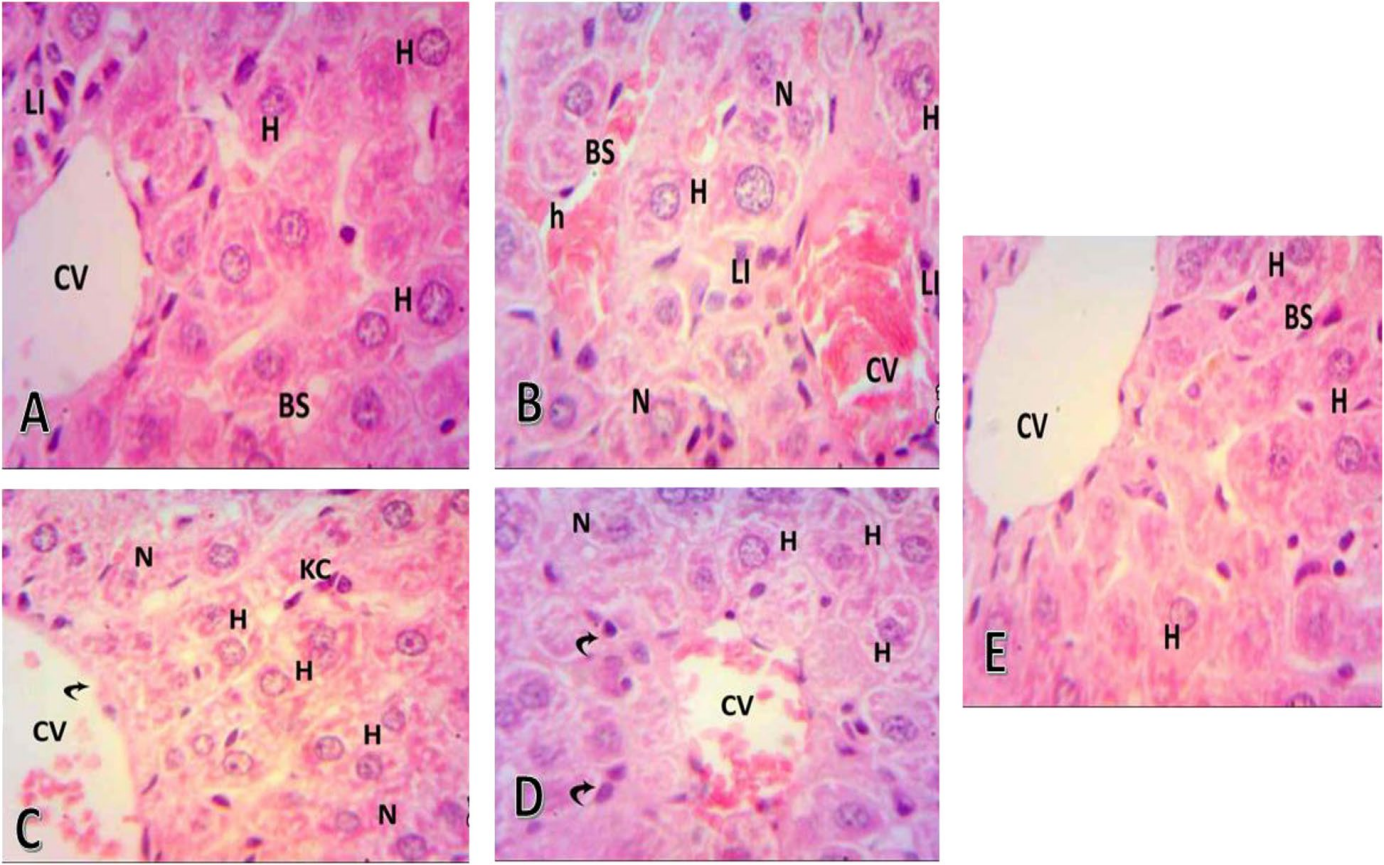
Histopathological sections of liver tissues from each mice group. A, Negative control group, showing the normal histological structure of the liver including central vein (CV), hepatocytes (H), blood sinusoids (BS), and lymphocytic infiltration (LI). B, positive control group, showing disorganized liver parenchyma including central vein highly congested with blood, extravasated hemorrhage, hepatocytes (H) with extensive cytoplasmic vacuolization for necrosis (N), dilated and congested blood sinusoids (BS), intensive lymphocytic infiltration (LI) surrounding destructed bile ductules (BD). C, Oxaliplatin treated mice, showing organized hepatocytes (H), with necrotic (N) regions, but a central vein (CV) still congested with few damaged erythrocytes, D, free CDA showing well organized hepatocytes (H) with vesicular nuclei, congested central vein (CV) with few damaged erythrocytes. E, CDA-Dextran conjugates showing well-organized hepatic parenchyma in hepatic strands including hepatocytes (H), still necrotic a clear central vein (CV) with intact body wall, Blood sinusoids (BS) appeared nearly similar to the control

The pharmacokinetic properties of free and CDA-dextran conjugates were assessed in vivo using male mice. After the 5th day of all the treatments, the mice were sacrificed, and the biochemical, histological, and immunohistochemical studies of mice, Ehrlich tumor cells (EAC), and liver were conducted. For the negative control group, the liver tissues formed of central veins appeared normal surrounded by rows of polyhedral hepatocytes with central nuclei and eosinophilic cytoplasm (Fig. 6). For the positive control mice, incubated for 5 days till the tumor size reached about 50 mm^3^, inoculated with 1 μM of 5-FC. From the photograph of the liver tissue (Table 7), a dilated congested central vein surrounded by lobules of hepatocytes with central nuclei and eosinophilic cytoplasm was observed. The lobules of hepatocytes are separated by dense fibrous bands and heavy aggregation of chronic inflammatory cells going towards necrosis and dilated and congested blood sinusoids were observed. An intensive lymphocytic infiltration surrounded by destructed bile ductless, disorganized liver parenchyma was observed, with the hepatic portal vein high congestion with blood and extravasated hemorrhage. For the treated mice with free-CDA, the liver tissue showed a markedly dilated congested central vein surrounded by rows of hepatocytes and aggregation of inflammatory cells, showing well organized hepatocytes with vesicular nuclei, but some of them still showing necrosis, and central vein still congested with few damaged erythrocytes (Fig. 7). For the CDA-dextran conjugates, the liver tissue showing central veins surrounding by rows of hepatocytes with scattered inflammatory cells. The liver sections have well-organized hepatic parenchyma in hepatic strands including hepatocytes. Bile ductless appeared organized with few lymphocytic infiltrations, blood sinusoids appeared nearly similar to that of control ones. From the histopathological investigations, the negative control mice liver showed normal blood vessels and normal nucleus and central veins surrounded by rows of polyhedral hepatocytes in the center. Positive control mice liver showed dilated congested central vein surrounded by lobules of hepatocytes with central nuclei and eosinophilic cytoplasm, the lobules of hepatocytes were separated by dense fibrous bands and heavy aggregation of chronic inflammatory cells (Fig. 7). Interestingly, treatment with both free CDA and CDA-Dextran conjugates, a dramatic reduction to the most of the pathological alterations induced by EAC cells in mice, was reported. The prodrug 5-FC-CDA system showed a great enhancement in the histology of the liver. The CDA-Dextran conjugates displayed a typical normal appearance to the liver tissues as reflected by a normal array of the hepatic cords radiating from the central vein, the cytoplasm is intact with normal eosinophilia, and the nuclei are similar to negative control ones. From the histopathological examination, there was a diminishing in pathological structure to a great degree, towards normal intact histological structure, upon using the 5-FC prodrug and CDA-Dextran conjugates, compared to the oxaliplatin-based chemotherapy.

**Table 7:** Effect of Oxaliplatin, free CDA and CDA-Dextran conjugates on the survival time (percentage increase in life span (ILS) and T/C % (treated vs. EAC control), EAC solid tumor weight and volume and activity of Caspase-independent apoptotic markers (PARP-1 and AIF). All values are presented as mean ± SEM. Statistical analysis was performed by one-way ANOVA (F-test). Statistical significance was defined as a *p*-value ≤0.05 indicating significant difference. Treated groups versus EAC positive control group, * *P* < 0.05. Ns, refers to non significant

Parameters	Positive control (EAC bearing) without any treatments	EAC + Oxaliplatin	EAC + Free CDA	EAC + CDA-Dextran
ILS%	_	39.47	65.78	78.94
T/C%	_	139.47	165.78	178.94
EAC solid tumor weight (g)	1.65 ± 0.15	1.45 ± 0.25	1.4 ± 0.16	1.3 ± 0.33
EAC tumor volume (mm3)	2.67 ± 0.26	1.9 ± 0.49	1.48 ± 0.70	1.43 ± 0.07
PARP-1(pg/mg protein)	325 ± 26.13	740.3 ± 61.68^ns^	808.7 ± 12.33*	1549 ± 134.5*
AIF (pg/mg protein)	57 ± 3.05	170 ± 6.93 ^ns^	257.3 ± 6.17*	406.7 ± 45.96*

Values are represented as mean ± SEM, where *n* = 10

Treated groups versus EAC positive control group, * P < 0.05

ns = non significant

**Fig. 8:**
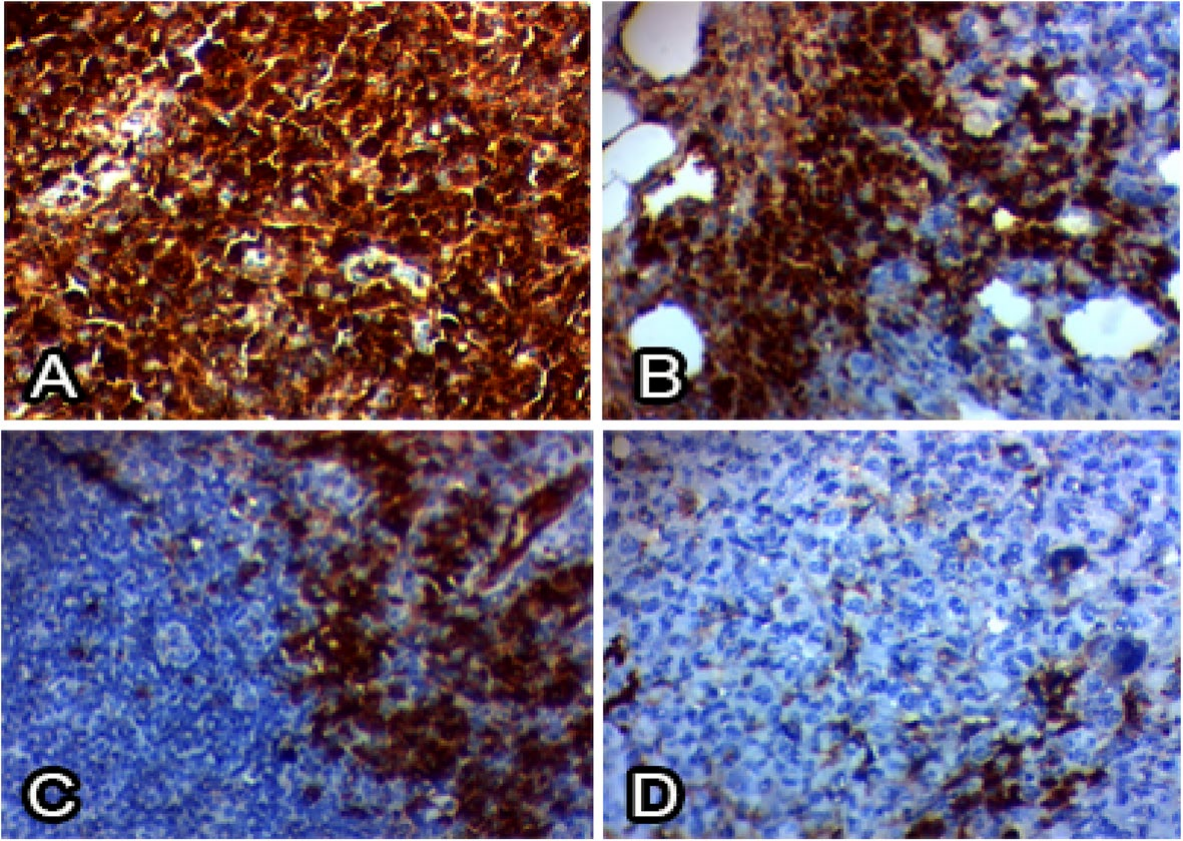
Immunohistochemical investigation of cyclin D1 in EAC solid tumor from the mice, A, positive control mice EAC. B, Oxaliplatin group. C, Free CDA treated mice, and D, CDA-Dextran conjugates treated mice

The biological response of EAC-mice regarding the tumor volume, weight, and Caspase-independent apoptotic markers (PARP-1 and AIF) in response to free enzymes was assessed. From the results (Table 8), the survival time on the life span (ILS) and T/C% (treated/control) were increased by two folds higher than the reference drug “oxaliplatin”, with obvious affordability for the efficiency of the 5-FC in presence of CDA-Dextran conjugates than the free CDA. The tumor volume and weight of EAC were reduced to 1.4 mm^3^, compared to 2.67 and 1.67 mm^3^ for control EAC-mice (untreated), that being more significant than the oxaliplatin-treated sample. Also, the CDA-Dextran conjugates displayed a slightly higher activity on activating the 5-FC prodrug than the free-CDA (Fig. 8). The Caspase-independent apoptotic markers PARP-1 and AIF, were increased by about 4.8 folds, i.e. 1549 pg/mg protein and 406.7 pg/mg protein, in response to the CA-Dextran conjugates. The titers of Caspase-independent apoptotic markers were improved in response to CDA treatment which is similar to the effect of oxaliplatin.

**Table 8:** Effect of Oxaliplatin, free CDA, and CDA-Dextran conjugates on Antioxidant and oxidative stress biomarkers measured in EAC solid tissue All values are presented as mean ± SEM. Statistical analysis was performed by one-way ANOVA (F-test). Statistical significance was defined as a *p*-value ≤0.05, indicating significant difference

Biochemical parameters	MDA (nmol/g tissue)	NO(umol/g tissue)	GSH (ug/g tissue)
Positive control (EAC bearing) without any treatments	182.7 ± 35.4	79.2 ± 4.5	67.70 ± 2.67
EAC + Oxaliplatin	91.6 ± 8.4*	68.3 ± 4.0*	70.64 ± 2.98*
EAC + Free CDA	61.9 ± 4.9*	60.9 ± 1.8*	73.66 ± 1.92*
EAC + Dextran-CDA	49.4 ± 4.8*	49.8 ± 4.2*	73.75 ± 1.877*

Values are represented as mean ± SEM, where n = 10

* indicates a statistically significant difference (P ≤ 0.05) compared to the positive control group

The biochemical responses of the experimental animals bearing EAC in response to treatment with the 5-FC-CDA system were determined, regarding the concentration of MDA, NO, and GSH (Table 8). The concentration of MDA in positive control mice bearing EAC was reduced by 3 folds in response to the prodrug 5-FC mediated by free CDA and CDA-Dextran conjugates, which is relatively similar to those reported for Oxaliplatin-treated samples. The free CDA and CDA-Dextran conjugates displayed the same response on modulating the concentration of NO and GSH of the mice of EAC, similar to the Oxaliplatin-treated mice. From the hematological results (Table 9), the titers of RBC and WBC were restored to 6.6–7.6 and 14.1–10.1, in response to the free CDA and CDA-Dextran conjugates, respectively, to be similar to negative control mice samples. The titer of WBC in EAC-mice was increased by two folds compared to the negative control. The levels of hemoglobin, RBC, WBC, and lymphocytes in EAC mice treated with the 5-FC-CDA system were restored to be very similar to those of the negative control, for both free CDA and CDA-Dextran conjugates.

**Table 9:** Effect of Oxaliplatin, free CDA and CDA-Dextran conjugates on hematological parameters (Hemoglobin level, RBC, WBC cells count) All values are presented as mean ± SEM. Statistical analysis was performed by one-way ANOVA (F-test). Statistical significance was defined as a *p*-value ≤0.05 indicating significant difference. a EAC control group versus negative control group. b Treated groups versus EAC positive control group

Parameters	Negative control	Positive control (EAC bearing) without any treatments	EAC + Oxaliplatin	EAC + Free CDA	EAC + Dextran-CDA
Hemoglobin (g/dl)	13.27 ± 1.90	5.93 ± 1.54* ^**a**^	9.38 ± 1.6* ^**b**^	8.53 ± 1.81* ^**b**^	10.81 ± 1.5* ^**b**^
RBC (million/m^3^)	8.90 ± 0.99	5.51 ± 0.22* ^**a**^	7.22 ± 0.27* ^**b**^	6.65 ± 0.25* ^**b**^	7.60 ± 0.26* ^**b**^
WBC (million/mm^3^)	9.03 ± 0.19	21.75 ± 0.38* ^**a**^	10.84 ± 0.26* ^**b**^	14.02 ± 0.38* ^**b**^	10.02 ± 0.34* ^**b**^
lymphocytes	71.18 ± 0.49	36.67 ± 0.60* ^**a**^	57.67 ± 0.49* ^**b**^	54.17 ± 0.70* ^**b**^	61.50 ± 0.67* ^**b**^

Values are represented as mean ± SEM, where n = 10

a EAC control group versus negative control group

b Treated groups versus EAC positive control group, * P < 0.05

## Discussion

Cancer chemotherapies have been challenged by drug resistance, low selectivity to the target tissues, and side effects on the human body [26]. Prodrug-mediating enzymes are one of the sophisticated strategies to overcome these challenges. CDA has been used for mediating the 5-FC into 5-FU, CDA derived from yeast had more affordable kinetic properties than bacterial one, however, the structural and thermal stability was the challenge for clinical trials [27]. Thus, exploring thermostable CDA from fungi was the objective. *A. niger* was the highest CDA producer, consistently for CDA production from yeasts [13], *A. fumigatus* [12], *A. parasiticus, A. niger*, *A. flavus*, *A. tamarii,* and *A. ustus* [13]. CDA was purified from *A. niger* by ion-exchange chromatography and gel-filtration [16, 28–30] by ~ 15.47 folds and yielded 40.02%. Consistent results using the same protocols were assessed for CDA from *A. fumigatus* [12], *Salmonella typhimurium* [31], *E. coli* [12], and *A. parasiticus* [13]. The molecular homogeneity of purified *A. niger* CDA was assessed, showing a single band of molecular mass 48 kDa and 98 kDa, on SDS-PAGE and native-PAGE, revealing the CDA homodimeric identity. Consistently, the molecular mass of *E. coli* CDA was 48 kDa [12], and heterodimers of 35 and 46 kDa subunits [32]. The molecular mass of *A. fumigatus* CDA was 32 kDa [12].

Enzyme modification by dextran conjugation has been considered one of the most powerful technologies for increasing the enzyme structural stability and catalytic efficiency by reducing their antigenicity and proteolysis [23, 25, 29, 33]. The purified CDA from *A. niger* was conjugated with activated dextran [34], to increase their structural stability and catalytic efficiency. Similar studies for the modification of therapeutic enzymes by dextran conjugation [25, 29, 33] and polyethylene glycol (PEG) [30, 35, 36] were reported. The conjugation yield of CDA with dextran was 57.54%, with overall activity of 219.9 compared to 382.2 μmol/min/mg of the free CDA. The modification percentage of the surface amino groups of CDA upon dextran conjugation was 41.6%, assuming the multiple interferences of activated dextran aldehydes with CDA surface primary amino groups of lysine, glutamine, and asparagine. Similar results were reported for methionine γ-lyase conjugated with dextran [30]. The modification of free surface thiols was 88.8% assuming the slight interaction of the chemical modifiers with the surface thiol groups of the enzyme. From the proteolysis profiles, dextran conjugation shields the surface recognition proteolytic sites of CDA. The free and CDA-dextran conjugates retain 16.7 and 47%, respectively, of their initial activity in response to proteinase K treatment. The resistance of CDA-dextran to proteolysis suggests the shielding of more than 52% of the CDA surface proteolytic recognition sites, that being matched with the in silico proteolysis map. The free CDA and CDA-Dextran conjugates had the same response to reaction temperature at 37 °C which is consistent with CDA from different sources [12, 14]. CDA-dextran conjugates had a higher thermal stability than the free one as revealed from the *T*_*1/2*_, assuming the higher catalytic and structural stability of CDA upon dextran conjugation, with a significant decrease to the thermal denaturation rate (*Kr*). In response to reaction pH, precipitation pH, and pH stability, both free CDA and CDA-dextran conjugates gave the same catalytic response, with the highest activity at pH 7.0. The free and modified CDA had the same stability response to different pH, with maximum stability at pH 6.0 to 8.0 [22, 30]. Chemical inhibitors are one of the most reliable approaches for active site mapping of enzymes, predicting their functional moieties. Overall, CDA-dextran conjugates showed higher catalytic and conformational structure stability than the free ones in response to different inhibitors. Intriguingly, the activity of de-metallized CDA (apo-CDA) was reduced by ~ 39.3% compared to the control, and their activity was restored in response to KCl ensuring their metallo-proteineous nature, dependency on K^+^ ions as cofactors. The inhibition of CDA by the addition of Hg^+ 2^ and Cu^+ 2^ suggests the incorporation of surface reactive thiols of the enzyme [12, 14, 37]. Consistently, the active site domains of enzymes were predicted by different amino acid suicide analogues DTNB, guanidine thiocyanate, iodoacetamide, hydroxylamine, H_2_O_2,_ and MBTH [21, 22, 38]. Dextran conjugation of CDA stabilizes the conformational structure of CDA and increases the resistance to oxidation by H_2_O_2_ by 10 folds, ensuring the steric protection of CDA active site residue from oxidation by H_2_O_2_. The free CDA activity was significantly reduced by 5 folds compared to Dextran-CDA upon treatment by DTNB ensuring the prevention of L-cysteinyl from oxidation by DTNB [21, 23]. Both Dextran-CDA and free CDA retains about 37.5 and 9.54% of their initial activities, after hydroxylamine addition, confirming that dextran conjugation gave stabilizing effect on surface lysine residues [21, 23, 37]. Similar results were reported for CDA from *A. fumigatus* [15] in response to strong inhibition by DTNB, *E. coli* [12]. The CDA from *Chromobacterium violaceum* has been verified as a Calcium-dependent enzyme [39]. The affinity (*K*_*m*_) of the free and CDA-dextran conjugates to deaminate 5-FC was 0.22 and 0.15 mM, respectively. So, upon dextran conjugation, the CDA affinity to deaminate 5-FC was increased by 68%. The catalytic efficiency (*K*_*cat*_*/K*_*m*_) of CDA-dextran was increased by ~ 1.5 folds compared to the free CDA, ensuring the improved catalytic properties of CDA upon dextran conjugation. Similar results were reported for many enzymes conjugated with dextran as glucose oxidase [40], methionine γ-lyase [38], and arginine deiminase [16]. Coincidently, the catalytic affinity (*K*_*m*_) to deaminate cytosine and 5-FC was reported for CDA from *S. typhimurium* (0.7 mM) [14], *E. coli* (0.2 mM) [41], *E. coli* (0.2 mM) and *C. violaceum* (1.55 mM). Similar results for the affinity of CDA for deaminating cytosine, 5-flurocytosine, cytidine, and 5-methylcytosine were reported for the enzyme from *A. parasiticus* [39] and *A. fumigatus* [12]. The antiproliferative activity of the prodrug system 5-FC-CDA has been assessed. The 5-FC-CDA system displayed significant activity against the breast carcinoma MDA-MB-, followed by HepG-2 and PC-3 cells. The viability of HepG-2 and PC-3 cells were reduced by about 50% in response to the free and CDA-dextran conjugates, at the concentrations of 200 μg/ml of 5-FC. The activity of CDA-dextran conjugates was increased by 1.8 folds higher than the free CDA towards the tested tumor cell lines. The higher antitumor activity of CDA upon dextran conjugation might be ascribed to the acquired relative improvement in the catalytic efficiency, solubility, turnover number, and rate of enzyme-substrate complex formation by dextran residues, coincident results for enzyme modulation by dextran residues were reported [5–11, 15–17, 20]. The in vivo activity of free and CDA-dextran conjugates towards the EAC tumor by mediating the prodrug 5-FC has been evaluated. The CDA-Dextran conjugates had a normal appearance in the liver tissues as reflected by the array of hepatic cords, and intact cytoplasm with normal eosinophilia. Histopathologically, a strong restoration to the normal histological structure, upon using the 5-FC prodrug mediated by CDA-dextran, compared to the oxaliplatin-based chemotherapy. The survival time “life span (ILS)” of animals was increased by 2 folds than oxaliplatin, using a 5-FC system mediated by CDA-dextran conjugates. The tumor volume and weight of EAC were reduced by two folds compared to negative control EAC-mice. The Caspase-independent apoptotic markers PARP-1 and AIF were increased by about 4.8 folds, in response to the CDA-Dextran conjugates. The titers of Caspase-independent apoptotic markers PARP-1 and AIF were improved in response to the CDA treatment, that being similar to the effect of oxaliplatin treatment. The MDA levels in positive control mice bearing EAC was reduced by 3 folds in response to the prodrug 5-FC mediated by free CDA and CDA-Dextran conjugates, that being relatively similar to those reported for Oxaliplatin treated samples as reference drug. The titer of WBC in EAC-mice was increased by two folds compared to the negative control. The levels of hemoglobin, RBC, WBC, and lymphocytes in EAC-mice treated with the 5-FC-CDA system were restored to be similar to the negative control, in response to the free and CDA-Dextran conjugates treatment.

In conclusion, *A. niger* CDA was purified and structurally modified via covalent conjugation with dextran, and the biochemical properties of the free and CDA-dextran conjugates were assessed. Upon dextran conjugation, the structural and catalytic stability of CDA have been noticeably improved. The catalytic efficiency of CDA-dextran conjugates for deaminating 5-FC was dramatically increased compared to the free CDA. The antiproliferative activity of the prodrug 5-FC by conversion into 5-FU was dramatically increased with CDA-dextran conjugates, authenticating the improvement in the catalytic efficiency, solubility, and turnover number of CDA upon dextran conjugation. From the in vivo studies using EAC-mice, the CDA-dextran conjugates displayed a higher anticancer activity as revealed from the EAC tumor size, weight, and biochemical markers Caspase-independent apoptotic markers PARP-1 and AIF. The free and CDA-dextran conjugates had no signs of toxicity in the normal animals as revealed from the hematological and biochemical parameters. The promising anticancer activity of the developed prodrug system of 5-FC mediated by thermostable *A. niger* CDA modified by dextran paves the way for further ongoing studies of the clinical trials and gene therapies.

## Materials and methods

### Isolation and screening of Cytosine deaminase-producing fungi

For isolation of CDA-producing fungi, different soil samples were collected from Monufia, Qaluobia, and Sharqia Governorates, Egypt, and used fungal isolation on Czapekʼs-Dox Agar medium, incubated for 7 days at 45 °C [42]. For screening for CDA-producing potency, a plug from each homogenous axenic fungal culture was inoculated into 50 ml Czapekʼs-Dox broth medium/ 250 ml Erlenmeyer conical flasks, incubated at 45 °C for 7 days. The fungal mycelia were collected by filtration, the pellets were washed with sterile distilled water, and their intracellular crude proteins were extracted [29, 30]. Five grams of each fresh fungal biomass were grinded into fine powder in liquid nitrogen, then dispensed to 5 ml Tris-HCl buffer (pH 7.0, 50 mM) with 1 mM phenylmethansulphonyl floride (PMSF), 10 μl β- mercaptoethanol and 1 mM CaCl_2_ [42–44]. The mixture was shaken for 10 min by vortex and centrifuged at 8000 rpm for 10 min at 4 °C. The supernatant was used as a crude source of CDA. The activity and protein concentration were determined.

### Cytosine deaminase (CDA) activity

The deamination activity of CDA was assayed as described by Sakai et al. [44, 45] with slight modifications. The reaction mixture contains 100 mM cytosine and 0.5 ml of enzyme in Tris-HCl buffer (pH 7.0, 50 mM) in 1 ml total volume. Blanks of enzyme and substrate were prepared separately. The reaction was incubated for 20 min at 37 °C and stopped by 10% TCA. The concentration of released uracil was assessed at 286 nm using blanks of cytosine and enzyme separately as baselines. Authentic concentrations of uracil were measured at the same conditions, and used for the calculation of enzymatic activity. One unit of CDA was expressed by the amount of enzyme releasing 1 mM of uracil from cytosine per min under standard assay conditions.

The protein concentration was measured by Folin’s reagent [46], compared to the authentic concentration of bovine serum albumin.

### Morphological and molecular identification of the potent fungal isolates

The potent CDA-producing fungal isolate was identified based on their morphological features according to the universal keys [22, 23, 47–50]. The identification of the potent fungi was confirmed from the sequence of internal transcribed spacers (ITS) region [24, 51, 52]. Briefly, 0.2 g of the mycelia was pulverized in liquid nitrogen, vigorously homogenized, 500 μl of extraction buffer (2% CTAB, 2% PVP40, 0.2% 2-mercaptoethanol, 20 mM EDTA, 1.4 M NaCl in 100 mM Tris-HCl, pH 8.0) was added. Genomic DNA (gDNA) was used as a PCR template with primers ITS4 5′-GGAAGTAAAAGTCGTAACAAGG-3′ and ITS5 5′-TCCTCCGCTTATTGATATGC-3′. The PCR reaction contains 10 μl of 2x master mixture (i- Taq™, iNtRON Biotech.), 2 μl gDNA, and 1 μl of each primer in 20 μl total volume. The amplicons were analyzed with 1% agarose gel in 1× TBE buffer using a DNA marker, purified, and sequenced. The obtained ITS sequences were searched using the BLAST tool with non-redundant sequences on the NCBI database. MEGA X software package was used for multiple sequence alignments, the FASTA sequences were imported, and aligned by the Clustal W algorithm [53], then the phylogenetic relatedness of the target sequences was constructed with 250 bootstrap replication [54].

### Purification, molecular subunit structure, and peptide fingerprint of CDA from *a. niger*

The potent fungus was grown on Czapek’s-Dox, mycelia were collected, and washed in sterile Tris-HCl buffer (50 mM, pH 7.0). Fungal pellets (50 g) were grinded into fine powder in liquid nitrogen and suspended in 50 ml Tris-HCl buffer of 1 mM EDTA, 1 mM PMSF, 1 mM β-mercaptoethanol and 1 mM CaCl_2_. After homogenization, the mix was shacked vigorously by vortex for 10 min then centrifuged for 10 min at 10,000 rpm to remove the cell debris. Dialyzer membrane of 20 kDa cut-off (Cat#546–00051) was used to concentrate the crude protein against PEG6000 [55]. The concentrated CDA preparations were further purified by gel-filtration and ion-exchange chromatographic techniques [56, 57] The most active fractions were collected, based on their activity for the subsequent biochemical characterization, and molecular mass homogeneity by SDS-PAGE analysis. The homogeneity and molecular subunit structure of the purified CDA were assessed by SDS-PAGE [58], normalizing to authentic protein marker. While, the molecular mass of the entire purified CDA was assessed by native-PAGE, without SDS on the sample and running buffers [23, 30].

The peptide fingerprint of the purified CDA was analyzed by the Liquid Chromatography-Tandem Mass Spectrometry of nanospray ionization (LC-MS/MS) at the Proteomics and Metabolomics Facility Core, 57,357 Children’s Cancer Hospital Foundation, Egypt. The SDS-PAGE gel band containing the putative CDA was excised, grinded, and destained by 200 μl of 50 mM ammonium bicarbonate (AB)-acetonitrile (1:1 v/v) [23]. The excess acetonitrile was removed by vacuum, the gel was re-swelled in 100 mM AB with 10 mM DTT for 30 min, followed by 100 mM AB with 50 mM iodoacetamide. The dried gel pieces were digested with trypsin for 12 h at 37 °C [30], the supernatant was pooled, dissolved in 100 μl of extraction buffer (5% formic acid/ acetonitrile, 1:2 v/v), and incubated for 15 min at 37 °C. The peptides were desalted and analyzed by nanospray ionization with Triple-TOF 5600 hybrid mass spectrometer, interfaced by nano-scale RP-HPLC [33, 59]. A linear gradient of acetonitrile (ACN) buffer (5–60%) was used for peptide elution from the column to mass spectrometry, with independent acquiring MS/MS data from m/z for 50–2000 Da. The raw MS/MS data were extracted, and the peptides were identified by Protein Pilot 4.0 (ABSCIEX) normalizing to the proteome of *Aspergillus niger*.

### Conjugation of the thermostable *a. niger* CDA with dextran

Sodium periodate-activated dextran was cross-linked with the purified CDA for 24 h at 4 °C, in presence of trimethylaminoborane (0.15 M) [60]. The Schiff base developed from CDA reactive amino groups and dextran aldehyde groups were stabilized by reduction with 3 mg/ml sodium borohydride [43]. Different molar ratios of purified CDA (0.067, 0.135, and 0.203 mM) and activated dextran (40 mM) were examined. The activity of free CDA and Dextran-CDA conjugates were assessed by the standard assay. The immobilization yield (%) of CDA was expressed by the ratio of the activity of conjugated CDA to the free- CDA × 100.

### Modification of the surface reactive groups of *a. niger* CDA upon dextran conjugation

The total surface reactive amino groups of the free and Dextran-CDA conjugates were assessed by Ninhydrin reagent [61]. The free CDA and Dextran-CDA conjugates (1.0 mg/ ml) were amended with 100 μl of Ninhydrin reagent, boiled for 5 min, and the developed blue complex was measured at A_575_ nm, compared to controls of activated dextran. The total surface reactive amino groups were expressed by the intensity of developed color at A_575_ nm of Dextran-CDA conjugates to the free enzyme × 100. The total surface reactive thiols of free and Dextran-CDA conjugates were determined by Ellman’s reagent (DTNB) [62] with slight modifications [24, 30, 43]. The enzyme (free and conjugated) were amended with 100 μl of 10% SDS, incubated for 10 min, and then 50 μl of 5,5′-dithiobis-(2-nitrobenzoic acid) (DTNB) (25 mM) was added, vortex and incubated for 30 min. The intensity of the developed yellow complex was measured at A_420_ nm, and the modification ratio of the surface thiols of CDA was expressed by the color absorbance of CDA-dextran conjugates to the absorbance of free CDA × 100.

### Proteolytic mapping of free and dextran-CDA conjugates

The structural stability of the free and CDA-dextran conjugates in vitro in response to proteolysis by proteinase K and trypsin were assessed [22, 23]. The native and CDA-dextran conjugates (0.8 mg/ml) were incubated with Trypsin and proteinase K (10 μmol/ min/mg) at 37 °C for 1 h, then PMSF (1 mM) was added to stop the proteolytic activity, and the remaining CDA activities were assessed by the standard assays.

### Biochemical properties of the free and dextran-CDA conjugates

The optimal reaction temperature of the free and CDA-Dextran conjugates was assessed by incubation of the reaction at 30, 37, 45, 50, and 55 °C, and the enzyme activity was determined by the standard method. To determine the enzyme thermal stability, enzymes were pre-incubated without substrate at different temperatures as 30, 37, 45, 50, and 55 °C, and the residual activities of the enzymes were assessed after 30, 60, 90, and 120 min by standard assay [22–25]. The enzyme thermal kinetic parameters such as half-life time (*T*_*1/2*_), thermal inactivation rate (*Kr*), and stabilization folds were assessed [22]. The effect of reaction pH (3.0–10.0) on the activity of free and Dextran-CDA conjugates was assessed in citrate-phosphate buffer (50 mM, pH 3.0–5.0), and Tris-HCl buffer (50 mM, pH 6.0–10.0), the reactions were incubated, and enzyme activities were determined. The pH stability was assessed by pre-incubating the enzyme at different pH (3.0–10.0) at 4 °C for 1.5 and 3.0 h, then measuring their residual activities by the standard assay [24, 25]. The pH precipitation profile of free CDA and Dextran-CDA conjugates were assessed by incubating the enzymes at a pH range (3.0 to 10.0) at 4 °C for 24 h, then centrifugation for 10 min at 10,000 rpm, and the precipitated proteins were collected and measured by Folin’s reagent [46]. The isoelectric point (*p*I) was defined by the pH at which maximal protein precipitation was obtained [56, 57]. The effect of inhibitors on the activity of free and Dextran-CDA conjugates was evaluated. The enzymes were desalted by dialysis against Tris-HCl buffer (pH 7.0, 50 mM) with 1 mM EDTA for 2 h. Different cations such as K^2+^, Ba^2+^, Fe^3+^, Hg^2+^, Ca^2+^, Al^3+^, k^+^, Na^+^, and Cu^2+^ were added to the enzymes at 1 mM concentration for 2 h at 4 °C, the substrate was added, and the activity of enzymes was measured by the standard assay. The effect of different suicide amino acid reactive analogues “hydroxylamine, iodoacetamide, guanidine thiocyanate, 5,5’-dithiobis-(2-nitrobenzoic acid) (DTNB), 3-methyl-2-benzothiazolinone hydrazone (MBTH) and hydrogen peroxide (H_2_O_2_)” on the activity of free CDA and Dextran-CDA conjugates were measured, the mixtures were incubated at 4 °C for 2 h, then the residual activity of enzymes was measured.

### Substrate specificity and kinetic parameters of the free and CDA-dextran conjugates

The affinity of free and modified enzymes towards various substrates; L-asparagine, L-tyrosine, L-arginine, L-methionine, L-cysteine, L-phenylalanine, L-glycine, and L-tryptophan has been evaluated comparing to cytosine as standard substrate at a final concentration (10 mM). The affinity of free and modified CDA for deamination of 5-flurocytosine as substrate was assessed [63, 64], and the concentration of released 5-flurouracil was calculated from their authentic concentrations at the same conditions. The enzyme kinetic properties such as Michalis-Menten constant (*K*_*m*_), maximum velocity (*V*_*max*_), turnover number (*K*_*cat*_), and catalytic efficiency (*K*_*cat*_*/K*_*m*_) of the free and CDA-dextran conjugates were determined by the GraphPad Prism Software Package [25, 30].

### In vitro anticancer activity

The antiproliferative activity of the native and CDA-Dextran conjugates was evaluated towards different tumor cell lines; liver carcinoma (HepG-2), breast carcinoma (MDA-MB-231), and prostate cancer (PC-3) regarding the normal cells Oral Epithelial cells (OEC) with 3-(4,5-dimethylthiazol-2-yl)-2,5-diphenyl tetrazolium bromide (MTT) assay [65]. The 96-well plate was seeded with 10^3^ cells/well and incubated overnight at 37 °C in a CO_2_ incubator, the prodrug 5-fluorocytosine was amended with different concentrations (0, 10, 20, 30 μg/ml)/ well, and the plate was incubated for 5 h, then different concentrations of each enzyme was added, then incubation was continued for 48 h at the same condition. The MTT reagent was added, and the developed purple formazan complex was measured at λ_570_ nm. The IC_50_ value was expressed by CDA activity reducing the initial number of tumor cells by 50%, regarding phosphate buffered saline.

### In vivo pharmacokinetics of native and CDA-dextran conjugates

The pharmacokinetic properties of the free and dextran conjugated enzymes were determined using male mice (25 g of 30 days old). The in vivo experiments were performed according to the guidelines of the Institutional Animal Care and Use Committee of the Faculty of Medicine, Zagazig University, and NIH guidelines under protocol 15–08-263. Prior to injection, the mice were acclimatized for 5 days. The experimental mice were grouped into five groups: 1- Negative control, mice free from Ehrlich Ascites Carcinoma (Ehrlich cells, EAC), 2- positive control, mice subcutaneously injected with 2.5 × 10^6^ of EAC cells, incubated for 5 days till the tumor size reached about 50 mm^3^, inoculated with 1 μM of 5-fluorocytosine (5-FC), 3- Native-CDA group, the eight days post-inoculated mice of EAC with 5-FC, were injected with single dose of the free CDA, 4-CDA-Dextran conjugates group, the eight days EAC post-inoculated mice with 5-FC were injected with a single dose of CDA-dextran conjugates. 5- Native-CDA treated samples, the eight days EAC post-inoculated mice without 5-FC, were injected with a single dose of native CDA without 5-FC. 6-CDA-Dextran treated samples, the eight days EAC post-inoculated mice without 5-FC, were injected with a single dose of CDA-dextran. At the end of the experiment, the mice were anesthetized with urethane (1 g/kg body weight) and sacrificed by cervical dislocation [66–69]. The remaining animals (5 in each group) were kept for evaluating the survival percentage (life span prolongation). Blood samples were withdrawn by cardiac puncture from all animal groups in tubes containing EDTA for hematological assays. Hemoglobin (HB), counts of white blood cells (WBCs), and red blood cells (RBCs) were analyzed by the standard automated assay.

The Ehrlich solid tumors were harvested from each mouse and rinsed with saline and various biochemical parameters, and histopathological and immunohistochemical analyses were conducted. For the biochemical parameters, the activity of poly [ADP-ribose] polymerase 1(PARP-1) was assessed using Mouse Poly [ADP-ribose] Polymerase 1 (PARP) ELISA Kit (Cat # MBS918279). The titer of apoptosis-inducing factor (AIF) was determined by Mouse AIF ELISA kit (Cat. # EM0826). The concentration of malondialdehyde (MDA), nitric oxide (NO), and reduced glutathione (GSH) were determined by the Biodiagnostic Kit.

The tumor volume and weight were analyzed: the tumor volume was measured by the caliper.

The volume was expressed in mm^3^ using the formula V = 0.5a x b^2^, where a and b are the short and long diameters, respectively.

The life span prolongation was determined according to the formula:

Increase in the life span ILS% = (T-C)/C × 100, Where T is the median survival time of treated mice, and C is the median survival time of positive control mice.

Part of the solid Ehrlich tumor along with liver tissue were fixed in 10% formalin embedded in paraffin and stained by hematoxylin and eosin (H and E) stain. Sections were microscopically examined and photographed. As well as, immunohistochemical staining analysis of the solid Ehrlich tumor was performed for investigating the cyclin D1 activity. Tissue sections (3–5 μm) were deparaffinized in xylene, slides were incubated for 10 min in 3% H_2_O_2_ to block endogenous peroxidase. Antibody binding was detected by Dako’s HRP Envision kit (DakoCytomation, Denmark). Tissue samples were incubated with primary antibody (Anti-Cyclin D1 antibody, ab16663, Abcam, UK, diluted in 1/100 PBS) for 1 h. The intensity of the staining areas was expressed as follows; grade-0, a total absence of staining or < 5% of cells stained; grade-1, mild to moderate nuclear staining (5–50% cells stained); grade-2, strong nuclear staining (> 50% cells stained).

### Deposition of the fungal isolate

The sequence of the most potent CDA CDA-producing fungal isolates *Aspergillus niger* was deposited into Genbank with accession # MW332264.1.

## Statistical analysis

Results were expressed as mean of triplicates ± standard deviation and the data was analyzed using student *t*-tests and One-way ANOVA in GraphPad Prism version 5 for Windows, “www.graphpad.com”.

## Supplementary Information

**Additional file 1: Table S1.** Screening for cytosine deaminase producing fungi. **Table S2.** Substrate specificity of free and Dextran-CDA conjugates. **Fig. S1.** PCR products of ITS region of A. niger (A.n.) and *A. fumigatus* (A.f) on 1.4% agarose gel using genomic DNA as PCR template. **Fig. S2.** SDS-PAGE profile of the purified and crude CDA from Aspergillus niger (A) and *A. fumigatus* (B). C, Is the native-PAGE profile of the purified CDA from *A. fumigatus* (Lane 1) and A. niger (Lane 2).

## Data Availability

All the data are available in the manuscript. The ITS sequence of *A. niger* as a potent CDA-producer was deposited on Genbank with accession# MW332264. https://www.ncbi.nlm.nih.gov/nuccore/1939997834.
