# Supplementary material for: Aspergillus Niger thermostable Cytosine deaminase-dextran conjugates with enhanced structure stability, proteolytic resistance, and Antiproliferative activity
Source: BMC Microbiol. 2023 Jan 10;23:9. doi: 10.1186/s12866-023-02754-8 (PMC9830863; doi:10.1186/s12866-023-02754-8)
Supplement: Supplementary file 1 — Additional file 1: Table S1. Screening for cytosine deaminase producing fungi. Table S2. Substrate specificity of free and Dextran-CDA conjugates. Fig. S1. PCR products of ITS region of A. niger (A.n.) and A. fumigatus (A.f) on 1.4% agarose gel using genomic DNA as PCR template. Fig. S2. SDS-PAGE profile of the purified and crude CDA from Aspergillus niger (A) and A. fumigatus (B). C, Is the native-PAGE profile of the purified CDA from A. fumigatus (Lane 1) and A. niger (Lane 2). [file 12866_2023_2754_MOESM1_ESM.docx]

**Table S1:** Screening for cytosine deaminase producing fungi.

| **Isolate**  **Code** | **Fungal isolate** | **Deamination activity**  **(μmol/min)** | **Protein (mg/ml)** | **Specific activity**  **(μmol/mg/min)** |
| --- | --- | --- | --- | --- |
| S1 | *A. carneus* | **330.04 ± 60.29** | **10.77 ± 0.28** | **30.64** |
| S2 | *A. flavus* 1 | **352.50 ± 41.10** | **7.39 ± 0.01** | **47.70** |
| S3 | *Penicillium* sp *1* | **318.91 ± 48.95** | **9.09 ± 0.05** | **35.08** |
| S4 | *A*. *ochraceous 1* | **336.77 ± 24.01** | **10.00 ± 0.10** | **33.68** |
| S5 | *A. terreus* | **309.08 ± 56.22** | **10.03 ± 0.06** | **30.82** |
| S6 | *A. flavipes* | **318.80 ± 29.30** | **9.82 ± 0.05** | **32.46** |
| S7 | [*A. nidulans*](http://drfungus.org/knowledge-base/aspergillus-nidulans/) | **344.54 ± 34.81** | **11.57 ± 0.24** | **29.78** |
| Q1 | *A. fumigatus 1* | **397.81 ± 26.01** | **8.33 ± 0.16** | **47.76** |
| Q2 | *A*. *ochraceous* 2 | **367.12 ± 37.23** | **8.25 ± 0.08** | **44.50** |
| Q3 | *P. notatum* | **172.55 ± 42.92** | **7.89 ± 0.01** | **21.87** |
| Q4 | *Fusarium oxysporum* | **159.87 ± 16.05** | **4.98 ± 0.27** | **32.10** |
| Q5 | [*P. expansum*](https://www.sciencedirect.com/topics/agricultural-and-biological-sciences/penicillium-expansum) | **254.32 ± 23.07** | **6.62 ± 0.01** | **38.42** |
| Q6 | *Aspergillus* sp 1 | **215.68 ± 22.33** | **8.01 ± 0.01** | **26.93** |
| Q7 | *Penicillium* sp 2 | **197.07 ± 56.28** | **8.54 ± 0.07** | **23.08** |
| Q8 | *Fusarium* sp | **186.94 ± 43.01** | **8.22 ± 0.05** | **22.74** |
| Q9 | *A. awamori* | **257.73 ± 15.06** | **6.79 ± 0.05** | **37.96** |
| Q10 | *A. niger 1* | **334.86 ± 82.66** | **7.77 ± 0.02** | **43.10** |
| M1 | *A. carneus* | **205.64 ± 30.70** | **6.40 ± 0.03** | **32.13** |
| M2 | *A. flavus 2* | **283.50 ± 23.04** | **6.45 ± 0.05** | **43.95** |
| M3 | *A. parasiticus* | **334.86 ± 12.90** | **7.37 ± 0.02** | **45.43** |
| M4 | *Penicillium* sp 3 | **220.26 ± 24.13** | **7.66 ± 0.01** | **28.75** |
| M5 | *A. fumigatus 2* | **195.95 ± 5.66** | **8.17 ± 0.03** | **23.98** |
| M6 | *A. flavus* 3 | **189.19 ± 21.09** | **7.47 ± 0.14** | **25.33** |
| M7 | *Aspergillus* sp 2 | **207.41 ± 36.80** | **7.12 ± 0.04** | **29.13** |
| M8 | *A. flavus* 4 | **254.85 ± 38.70** | **8.53 ± 0.06** | **29.88** |
| M9 | *A. niger 2* | **375.68 ± 31.88** | **6.87 ± 0.08** | **54.68** |

**Table S2:** Substrate specificity of free and Dextran-CDA conjugates

| Substrate | Substrate conc. (mM) | Free-CDA Relative activity% | Conjugated - CDA Relative activity % |
| --- | --- | --- | --- |
| Cytosine | 10 | 100.00 | 100.00 |
| L-Arginine | 10 | 0.00 | 3.33 |
| L-Asparagine | 10 | 0.00 | 0.00 |
| L-Tyrosine | 10 | 0.00 | 3.33 |
| L-Methionine | 10 | 5.83 | 0.00 |
| L-Cysteine | 10 | 0.00 | 5.80 |
| L-Glycine | 10 | 0.00 | 0.00 |
| L-Phenylalanine | 10 | 9.17 | 4.35 |
| L-Tryptophan | 10 | 4.17 | 9.28 |


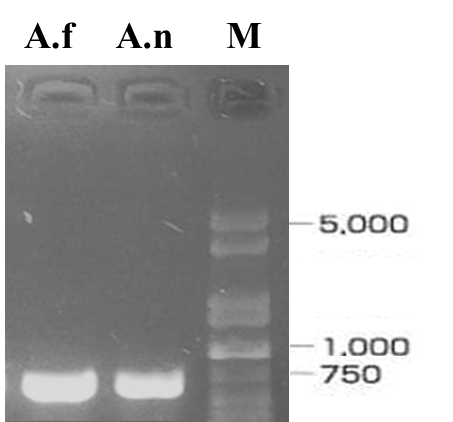


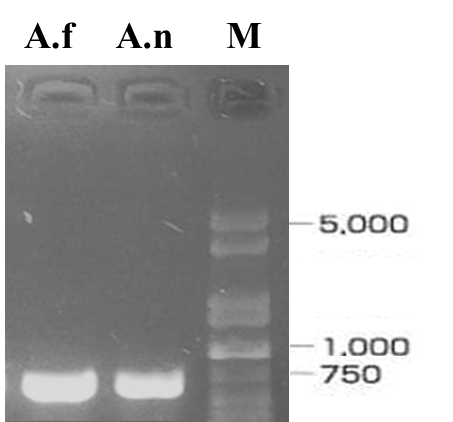


**Fig. S1: PCR products of ITS region of *A. niger* (A.n.) and *A. fumigatus* (A.f) on 1.4 % agarose gel using genomic DNA as PCR template.**


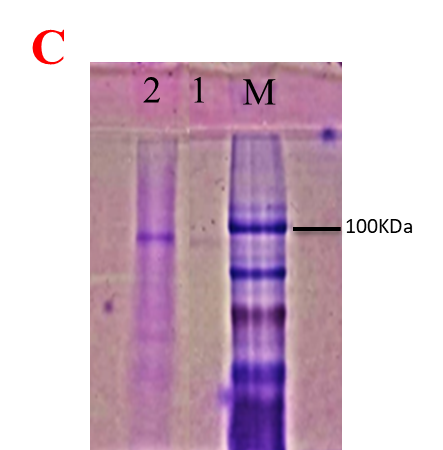

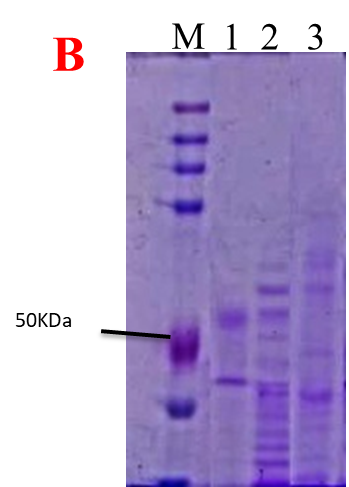

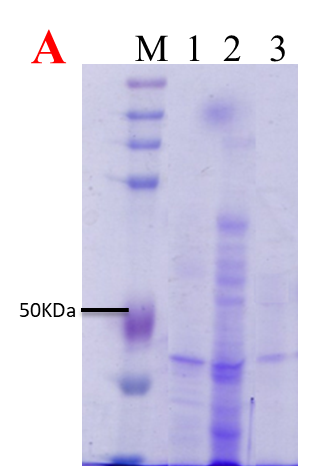


**Fig. S2. SDS-PAGE profile of the purified and crude CDA from *Aspergillus niger* (A) and *A. fumigatus* (B). C, Is the native-PAGE profile of the purified CDA from *A. fumigatus* (Lane 1) and *A. niger* (Lane 2).**
